# *i*GNM 2.0: the Gaussian network model database for biomolecular structural dynamics

**DOI:** 10.1093/nar/gkv1236

**Published:** 2015-11-17

**Authors:** Hongchun Li, Yuan-Yu Chang, Lee-Wei Yang, Ivet Bahar

**Affiliations:** 1 Department of Computational and Systems Biology, School of Medicine, University of Pittsburgh, PA 15213, USA; 2 Institute of Bioinformatics and Structural Biology, National Tsing-Hua University, Hsinchu 300, Taiwan

## Abstract

Gaussian network model (GNM) is a simple yet powerful model for investigating the dynamics of proteins and their complexes. GNM analysis became a broadly used method for assessing the conformational dynamics of biomolecular structures with the development of a user-friendly interface and database, *i*GNM, in 2005. We present here an updated version, *i*GNM 2.0 http://gnmdb.csb.pitt.edu/, which covers more than 95% of the structures currently available in the Protein Data Bank (PDB). Advanced search and visualization capabilities, both 2D and 3D, permit users to retrieve information on inter-residue and inter-domain cross-correlations, cooperative modes of motion, the location of hinge sites and energy localization spots. The ability of *i*GNM 2.0 to provide structural dynamics data on the large majority of PDB structures and, in particular, on their biological assemblies makes it a useful resource for establishing the bridge between structure, dynamics and function.

## INTRODUCTION

Several studies in the last decade have drawn attention to the significance of intrinsic dynamics as a major determinant of the mechanism of action of proteins and their complexes (1–5). Intrinsic dynamics refers to the conformational changes intrinsically favored by the 3-dimensional (3D) structure. These are equilibrium motions that maintain the native fold while allowing for concerted subunit or domain rearrangements (*global* motions) or for more localized conformational changes such as loop motions or side chain rotations (*local* motions), often relevant to biological function. These motions underlie the adaptation of biomolecules to their functional interactions and play an essential role in allosteric signaling (6). As a consequence, an important question is to assess which structural elements (e.g. residues, secondary structures, domains or entire subunits) undergo large fluctuations away from their mean positions (i.e. those enjoying high *mobility*), or which structural elements provide adequate *flexibility* to enable conformational changes (e.g. hinge-bending sites) that may be relevant to function. Furthermore, it is often of interest to determine which structural elements are subject to strongly correlated (or anticorrelated) motions, toward gaining insights into allosterically coupled regions. The Gaussian Network Model (GNM), introduced almost two decades ago (7,8) has served as an efficient, yet powerful, tool for addressing these questions, supported by the *i*GNM database (9) and its online computation server (10). GNM provides information on the size of motions of individual structural elements as well as the correlations between the motions of these elements. It has proven useful in a broad range of applications, e.g. for predicting the elastic modulus of protein nanofibrils (11), evaluating the coexistence of stability and flexibility in proteins (12), quantifying entropic contributions to binding free energy (13), assessing the significance of collective dynamics in the mechanochemical activity of enzymes (14), and identifying dynamically coupled domains and interdomain binding sites (15), to name a few. The basic idea behind the GNM is that folded structures, under native state conditions, have access to a spectrum of motions (or modes), which can be delineated by a simple description, an elastic network representation of structure. Adoption of an elastic network model (ENM) permits to take advantage of the established theory and methods of macromolecular statistical mechanics (16). The solid physical foundations as well as mathematical simplicity led to the broad usage of ENMs for efficient and accurate determination of collective dynamics using normal mode analysis (NMA) methods (2,17,18).

A crucial feature of the GNM is its ability to easily decompose the motions into a *spectrum of modes*, and extract *global* (low frequency, *slow* or soft) modes, or *local* (high frequency, *fast* or stiff) modes, similar to NMA but in a significantly simpler and more efficient way. The former group of modes usually underlies cooperative functional events including allosteric rearrangements, and the latter relates to energy localization and folding nuclei (19–24). Agreement between experimental data and GNM predictions consistently gave support to the utility of the GNM, beside its conceptual simplicity and computational efficiency. Examples of experimental data that have been used in benchmarking GNM predictions include X-ray crystallographic B-factors (25), H/D exchange data (26), NMR data (27), conformational variability derived from the principal component analysis (PCA) of ensembles of structures resolved in different forms for a given biomolecule (28) – protein (5,14,29) or RNA (30,31).

The observed utility of the GNM for identifying dynamically coupled domains led to the development of servers for predicting the hinge sites in biomolecular structures (32,33), building on earlier work for visualizing molecular motions (34). Notable efforts have been made for evaluating and disseminating collective modes of motions using ENMs and/or NMA (35–44), including the development of ENCoM server (45) for exploring the effect of mutations. Despite all these efforts, the DBs on ENM/NMA-based collective motions have been limited to a few studies such as *i*GNM (9) and *Pro*mode (37,42) DBs. In particular, *Pro*mode provides data on for 52 014 Protein Data Bank (PDB) (46) structures using an all-atom NMA in dihedral angle space. However, these are usually limited to single chains, or asymmetric units reported in the PDB; whereas for many applications, and in particular for multimeric systems, the dynamics of the biologically functional form, also called *biological assembly* (BA), is of interest.

We present in this study an updated version of *i*GNM, *i*GNM 2.0. The current version is a substantial advancement over the original *i*GNM DB developed in 2005. First, the total number of structures for which dynamics data are made available increased from 20 058 in version 1 to more than 100 000. Second, we took advantage of the improved techniques (Ajax, JQuery, HTM5, PHP and Highcharts) that enhanced the security and interoperability of the resource. Third, more results for each entry are reported compared to the earlier version, using interactive molecular viewers and charts. Fourth, *i*GNM 2.0 provides data not only for proteins, but for practically all types of PDB structures, including the complexes with DNA and RNA molecules or other substrates. Finally, GNM data are provided for the BA in the PDB, after assembling the biologically functional (usually multimeric) structure from the coordinates deposited for the asymmetric unit whenever applicable. The new database now provides access to pre-computed data on the dynamic properties of many supramolecular structures, which may help build plausible hypotheses for further studies.

## MATERIALS AND METHODS

### GNM and mode spectral analysis

**Figure 1.**
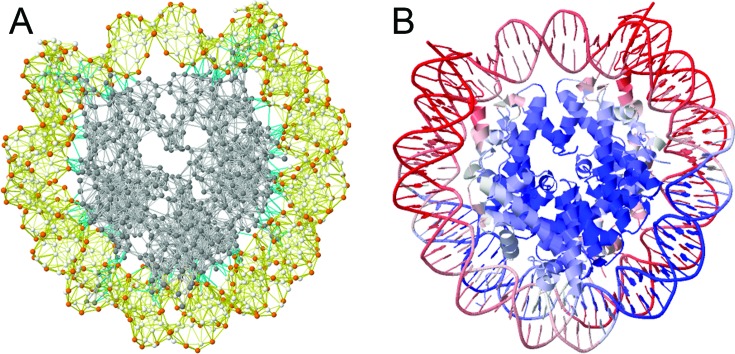
GNM representation of biomolecular structures and color-coded ribbon diagrams used in the *i*GNM DB, illustrated for the nucleosome core particle (PDB id: 1KX4). (**A**) The GNM representation consists of a series of nodes located at the positions of the C^α^-atoms (*gray*) for proteins, and at the P (*orange*), C4‘- and C2-atoms (*white*) for DNA/RNA. The nodes are connected by elastic springs, shown by *light-gray* (intramolecular, protein), *yellow* (intramolecular, DNA/RNA) or *cyan* (intermolecular) lines. (**B**) Ribbon diagram of the same structure, color-coded by residue square-fluctuations in the softest two modes computed by the GNM analysis. The colors vary from *red* (most mobile) to *blue* (most rigid).

In the GNM, the nodes are identified by the α-carbons (of amino acids for proteins) and P, C4’ and C2 atoms (of nucleotides for DNA/RNA molecules) (Figure 1); and the springs are placed between all pairs of nodes/residues within a first inter-residue coordination shell in folded structures – identified to be *r_c_* ≈ 7.0 – 7.5 Å for folded proteins (47). The connectivity of the network is defined by the Kirchhoff matrix, **Γ**. The off-diagonal elements of **Γ** are **Γ***_ij_* = **Γ***_ji_* = −1 if nodes *i* and *j* are within *r_c_*, and zero otherwise; and the diagonal elements represent the coordination numbers (or degrees) of the residues (nodes), found from **Γ***_ii_* = − **Σ***_j_* **Γ***_ij_* where the summation is performed over all elements *j, j*≠ *i*. Knowledge of **Γ** completely defines the network topology, and permits us to evaluate the intrinsically favored (or natural) modes of motion (relaxation) uniquely accessible to the structure. The ms fluctuations of residues (<Δ***R****_i_$\bullet$*Δ***R****_i_*> or <(Δ***R****_i_*)^2^> where Δ***R****_i_* is the change in the position vector of node/residue *i*) directly scale with the diagonal elements of **Γ**^−1^; and the cross-correlations between residue fluctuations scale with the off-diagonal elements, i.e.

(1) \begin{equation*} \lt \Delta {\boldsymbol{R}}_i \bullet \Delta {\boldsymbol{R}}_j \gt \sim [{\bf \Gamma} ^{ - 1} ]_{ij} \end{equation*}

The proportionality constant is *3k_B_T/γ*, where *k_B_* is the Boltzmann constant, *T* is the absolute temperature and *γ* is the force constant assumed to be uniform for all springs in the network. The value of *γ* does *not* alter the ‘distribution’ of fluctuations nor does it affect the *orientational* cross-correlations

(2) \begin{eqnarray*} C_{ij}^{orient} &=& \lt \Delta {\boldsymbol{R}}_i {\bullet}\Delta {\boldsymbol{R}}_j \gt /\,[ \lt (\Delta {\boldsymbol{R}}_i )^2 \gt \lt (\Delta {\boldsymbol{R}}_j )^2 \gt ]^{1/2} \\ \nonumber \ \ \ \ \ \ \ \ \ \ \ &=&[{\mathbf \Gamma} ^{ - 1} ]_{ij} /([{\mathbf \Gamma} ^{ - 1} ]_{ii} [{\mathbf \Gamma} ^{ - 1} ]_{jj} )^{1/2} \end{eqnarray*}

The fluctuation profile and the above cross-correlations are obtained without any parameters. Agreement with experiments without any adjustable parameter is the major strength of the GNM. Because the rows/columns of **Γ** are not independent, **Γ**^−1^ is the pseudo inverse obtained as

(3) \begin{equation*} {\mathbf \Gamma} ^{ - 1} = (3k_B T/\gamma ){\mathbf \Sigma} _k \lambda _k^{ - 1} {\boldsymbol{u}}_k {\boldsymbol{u}}_k^T = (3k_B T/\gamma ){\mathbf \Sigma} _k [\mathbf C]_k \end{equation*}

where the summation is performed over the *N-1* nonzero eigenvalues *λ_k_* of **Γ** and the corresponding eigenvectors ***u****_k_*. The eigenvector ***u****_k_* represents the normalized distribution of displacements for the *N* nodes along the principal/normal (mode) axis *k*, and the eigenvalue *λ_k_* scales with the square frequency of the fluctuations along this axis. The contribution

(4) \begin{equation*} [{\mathbf C}]_k = \lambda _k^{ - 1} {\boldsymbol{u}}_k {\boldsymbol{u}}_k^T \end{equation*}

of mode *k* to ms fluctuations or cross-correlations scales with *λ_k_*^−1^ such that the lowest frequency mode (*k* = 1, *λ_1_* ≤ *λ_2_* ≤ …. *λ_N-1_*) makes the largest contribution. Details on the derivations of GNM equations can be found in our previous work (48).

### Data set

**Figure 2.**
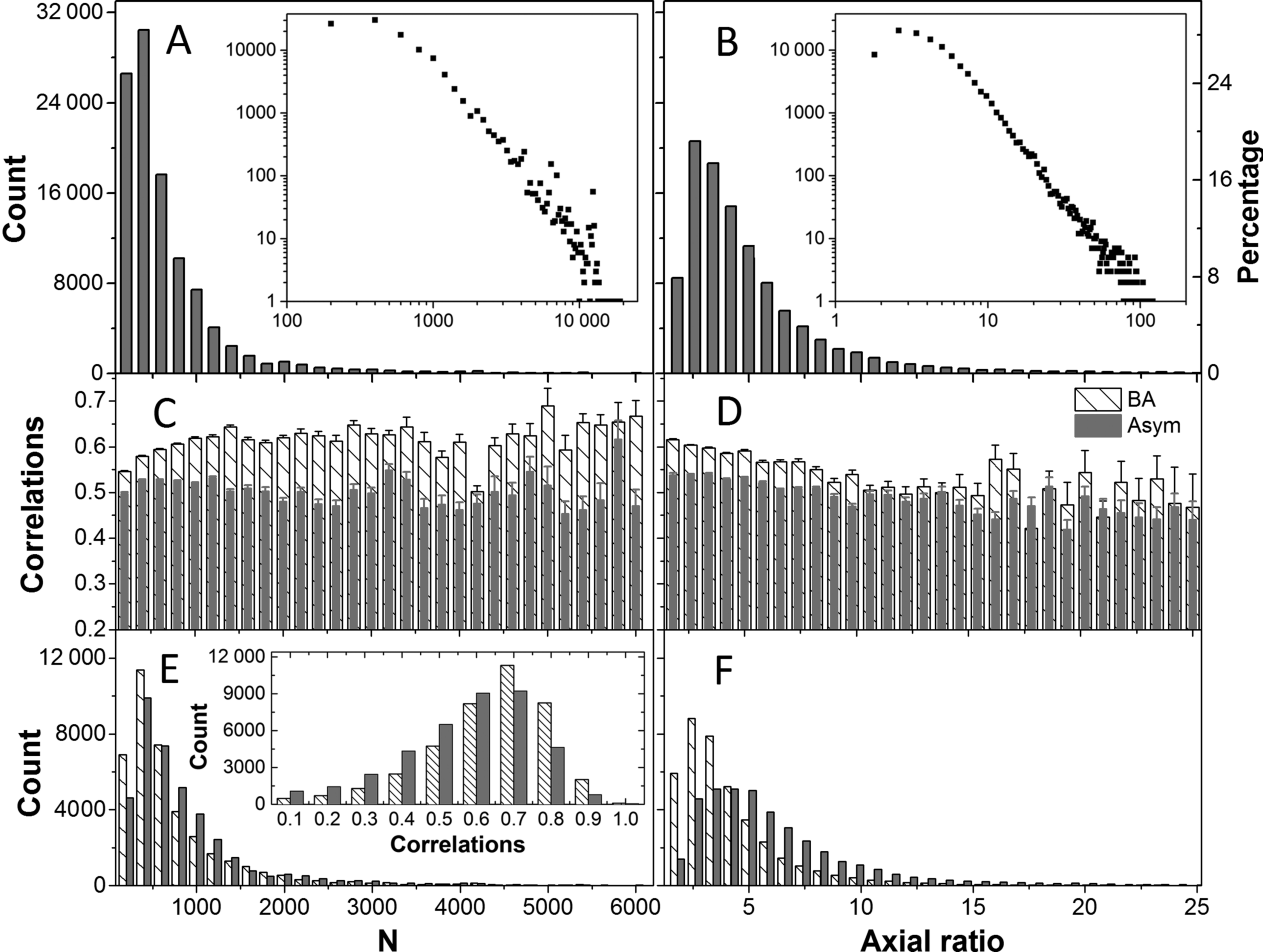
Distributions of the sizes and shapes of PDB structures in the *i*GNM 2.0 and correlation between experimental and theoretical mean-square fluctuation profiles. (**A**) Size distribution in terms of *N*, the number of nodes. For proteins, *N* is equal to the number of amino acids, for RNA/DNA it is 3 x number of nucleotides, each nucleotide being represented by three nodes. The size of the structures in the GNM DB varies in the range 12 ≤ *N* ≤ 20 872. The *left* and *right* ordinates display the count and percentage, respectively, based on bins of Δ*N* = 200. The logarithmic plot in the inset permits to view the distribution of larger structures. 13.9% of the structures in the *i*GNM 2.0 (14 899 out of 107 201) contain >10^3^ nodes. (**B**) The distribution of axial ratios, *a*. The counts (*left ordinate*) and percentages (*right ordinate*) refer to bins of size Δa = 0.8, starting from a = 1. Some of the structures are highly asymmetric (axial ratio ∼100). (**C**–**F**) Results for 39 505 PDB structures whose biological assembly (BA) is different from default structure reported in the PDBs (asymmetric unit, Asym). Panels (**C**) and (**D**) display the correlation coefficients (and their standard errors, shown by the error bars) between experimentally observed and GNM-predicted ms fluctuations, for the default PDB coordinates (*gray bars*) and the corresponding BAs (*dashed bars*), as a function of the size *N* (C) and axial ratio *a* (D) of the structures. Experimental data are based on the X-ray crystallographic B factors. Panels **E** and **F** display the corresponding counts, and the inset in E gives the distribution of correlations. A considerable increase in the level of agreement with experiments is achieved upon performing the analysis for the BA, rather than the default PDB file.

All the structures deposited in PDB as of June 30, 2015 were downloaded (109 457 of them) (46). For each of NMR structures, the first model among those deposited in the PDB, was used in GNM calculations. Likewise, the first BA files were used for those having multiple BA records in PDB. Structures containing less than 12 nodes or more than ∼20 000 nodes as well as those having data for C^α^-atoms only were filtered out, which led to 107 201 PDB files. The size and shape distributions of these structures are shown in Figure 2, respective panels **A** and **B**. The former shows the histogram as a function of the number of nodes, and the latter as a function of the axial ratio (i.e. the ratio of the largest principal axis to the smallest obtained by PCA of structural coordinates) (15). The *i*GNM 2.0 therefore contains information on the dynamics of biological assemblies of up to 2 × 10^4^ residues, and up to an axial ratio of ∼100.

### Inputs: query and searching functions

The *i*GNM 2.0 offers two options for searching the database. The first is to directly enter the PDB 4-letter id. The second is an advanced query function that permits users to search the database using one or more properties, such as the experimental method, the resolution of the structure (if applicable), the structure name (or title word), an author name, the release date, the residue count or molecular weight. Users may also search by entering dynamic features such as degree of collectivity of the GNM modes. The user is then directed to a relational table that includes all the PDBs entries that match the search. These entries can be sorted by features such as residue count or resolution. The relational tables can be exported as plain text file (tsv or csv format) or Excel file (xls or xlsx format).

## RESULTS

The *Results* page contains a J(s)mol window (*on the left*) illustrating the investigated structure (or its biological assembly, if applicable) color-coded by the square displacements of residues, and a panel of results (*on the right*). The panel contains seven clickable items described below, by way of an example, e.g. DNA/AlkB family demethylase complex (PDB id: 4NIH) (49).

**Figure 3.**
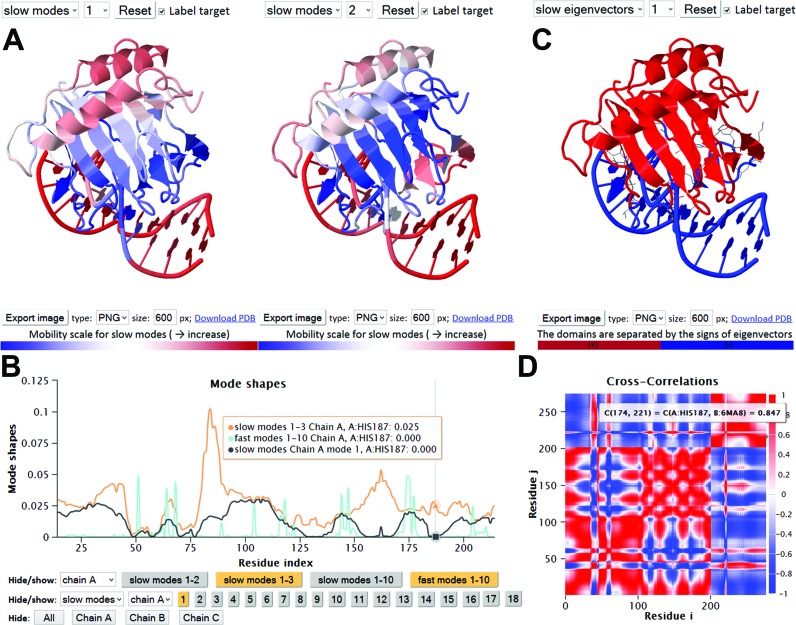
Results from *i*GNM 2.0 for DNA/AlkB family demethylase complex. Panel **A** displays the color-coded ribbon diagrams, from *red* (most mobile) to *blue* (most rigid) in the selected modes, rendered using JSmol. Panel **B** shows the shapes of selected modes (colored *orange* in the keys underneath) for chain A (demethylase): softest mode (slow mode 1, *black*), cumulative contribution of slow modes 1–3 (*orange*) and fastest 10 modes (*cyan*). Minima in the slow modes refer to key mechanical or chemical sites such as the hinge sites or the catalytic sites. These are held in place during the collective motions of the remaining parts. In this case, H187 is a catalytic residue. Peaks in the fast modes refer to centers of energy localization. (**C**) Domain separation obtained by mode 1. This mode separates the enzyme and DNA molecules indicating that the two molecules undergo anticorrelated motions in this most cooperative mode. (**D**) Orientational cross-correlations, associated with the slowest three modes. *Red* regions refer to residue pairs that move in the same direction (C*_ij_^orient^* > 0); *blue* regions refer to the pairs moving in opposite directions (C*_ij_^orient^* < 0), and uncorrelated pairs are shown in *white* (color-code bar on the *right*). Residue numbers along the axes refer to those of all chains ordered by chain index. Here, chains B and C are the two DNA strands, each of length 13, and chain A is the enzyme of 214 residues.

- **X-ray crystallographic B-factors (3D/2D)**. The X-ray crystallographic B-factors B*_i_* = (8π^2^ / 3) <(Δ***R****_i_*)^2^> provide a direct measure of the ms fluctuations of residues and provide an estimate of the correlation between experimental data and GNM predictions. The B-factors page is organized in two parts: the upper half displays two J(s)mol ribbon diagrams, one color-coded by the experimental B-factors (from *red*, most flexible; to *blue*, most rigid), and the second color-coded by the GNM-predicted ms fluctuation profile, and reports the correlation coefficient between the two sets of data; and the lower half displays the corresponding pairs of curves as a function of residue index for any selected chain. For 4NIH, the correlation coefficient is 0.80 and results are reported for three chains (a protein chain and two DNA chains).
  Table 1 lists the correlation coefficients between experiments and theory averaged over all 97 959 PDB structures resolved by X-ray in our data set. Results are presented for different subsets of PDB structures: Subset S1 refers to the cases where the PDB structure accessible by default (also called *asymmetric unit, Asym*) is identical to the BA. Those in subset S2 are not. They consist of two groups: S2B where the BA is constructed by assembling multiple copies of the default structure reported in the PDB, and S2A where the BA is a part of the default structure. Note that the consideration of the entire BA constructed by assembling multiple copies of the asymmetric units is essential to obtaining a higher correlation with experiments (see subset S2B, last row in Table 1). Figure 2 panels **C**–**F** provide more information on the correlation coefficients obtained for BAs (*dashed bars*) and Asym (*gray bars*). Supplementary Figure S1 shows the same results for the 97 959 X-ray structures included in the *i*GNM 2.0. We note that the agreement with experiments improves with decreasing asymmetry (or axial ratio) and increasing size.
- **Mode shapes (3D/2D)**. Similar to the B-factors page, the upper half of this webpage displays two color-coded ribbon diagrams. These help compare the mobilities of residues in two different modes (*GNM modes 1* and *2*, by default), as illustrated in Figure 3A. The lower half of the page displays the *mode shapes* (i.e. square displacements of residues driven by a given mode, plotted as a function of residue index). The ribbon diagram color code and mode shape for mode *k* are obtained from the diagonal elements of [**C**]*_k_* (see Equation 4). Results for both slow/soft modes and fast/stiff modes can be viewed. In the former case, the residue motions are (usually) uniformly-distributed across the structure (the modes are highly *collective*); in the latter, a number of sharp peaks appear in the mode shape (the modes are highly *localized*). Panel B in Figure 3 illustrates such selected modes.
- **Domain separations by dynamics (3D/2D)**. Each residue *i* moves in either the positive or negative direction along a given mode axis. The direction along mode *k* is given by the sign (+ or −) of the *i^th^* element of ***u****_k_* (each element corresponding to a residue or node). The subsets of residues moving in opposite directions are said to undergo *anticorrelated* movements in mode *k*. Each mode thus separates the structure into two subsets of residues that move in opposite directions (colored *red* and *blue* in Figure 3C). Note that in the global modes, residues in a given subset are spatially contiguous (they form coherent domains/subunits, etc.); whereas in the higher modes, they consist of multiple, more localized elements. Residues at the crossover regions between + and − directions define the interfaces between the anticorrelated domains in the global modes. The interface often includes a global hinge sites that plays a key mechanical role in enabling the relative movements of the domains. Likewise, key chemical residues (e.g. catalytic residues) whose precise (fixed) positioning is essential for activity usually lie at such interfacial regions, and as such they undergo minimal (if any) displacement in these modes minima (14).
- **GNM connectivity model (3D/2D)** page displays the topology of the network as an interactive 3D network model (Figure 1) or a 2D representation similar to a contact map.
- **Cross-correlations (3D/2D)** page displays the orientational correlations (C*_ij_^orient^*) between pairs of nodes, for the user-selected mode. Two maps are simultaneously displayed, with the second permitting to focus on selected regions of the former. Maps for customized subsets of modes can be calculated using the online calculation engine for *N* < 1000 nodes. Figure 3D illustrates the map for demethylase complex, based on the three slowest modes. The colors distinguish the correlated (*red*) and anticorrelated (*blue*) pairs of residues.
- **Collectivity (2D)** for a given mode *k* is a measure of the degree of cooperativity (between residues) in that mode, defined as (50)
  (5) \begin{equation*} {Collectivity}_k = \frac{1}{N}e^{ - \sum\limits_i^N {u_{k,i}^2 \ln u_{k,i}^2 } } \end{equation*}
  where, *k* is the mode number and *i* is the residue index. A larger collectivity value refers to a more distributive mode and *vice versa*. Usually soft modes are highly collective. Collectivity values are reported for soft modes that account for 1/10 of the overall dynamics.
- **Results in plain text**. All GNM results accessible in the above six output pages can be downloaded via HTTP, for further analysis and alternative visualizations. Modes at the low frequency end of the spectrum (the most favorable modes from energetic point of view) up to 40% of the spectrum are stored and can be retrieved for each PDB entry.

**Table 1.** Correlation between X-ray crystallographic B-factors and GNM-predicted mean-square fluctuation profiles^a^

Subset^b^	Count	Default PDB file (Asym)	Biological Assembly (BA)
S1	58 128	0.581 ± 6.8E-04	0.581 ± 6.8E-04
S2	39 505	0.518 ± 8.9E-04	0.589 ± 8.2E-04
S2A	23 343	0.522 ± 1.1E-03	0.575 ± 1.0E-03
S2B	16 162	0.513± 1.1E-03	0.610 ± 1.3E-03

^a^Results are reported as average correlations for the indicated subsets ± standard error.

^b^In the subset S1, the BA is identical to the default structure accessible at the PDB; Subset S2 consists of two subgroups, S2A and S2B; in S2A, the BA is a part of the default PDB (the asymmetric unit); in S2B, it is assembled from multiple copies of the whole/part of the default PDB using the transformation matrices reported in the PDB.

### Database architecture of *i*GNM 2.0

**Figure 4.**
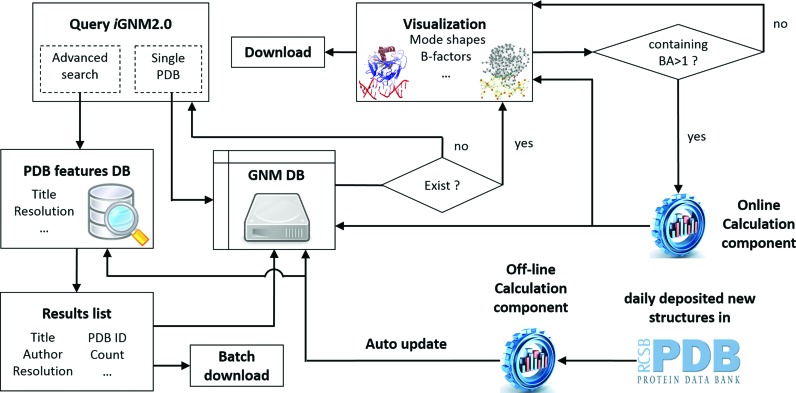
The architecture of the *i*GNM 2.0. Selected structural and dynamics features as well as experimental conditions of all the PDB structures are collected and stored in a ‘PDB features DB’, programmed with MySQL. *i*GNM 2.0 can be queried with a single PDB ID or customized search conditions using an advanced search component. The resulting list can be downloaded as a relational table and the corresponding GNM results files can be downloaded using a batch download script. The GNM results, in plain text and image format, can be downloaded via HTTP request from the GNM DB. The queried GNM results can be viewed from the visualization component with six interactive results pages constructed using HTML5, J(s)mol, Javascript, Jquery, Ajax and Highcharts techniques. Alternative BAs (if reported in the PDB) can be calculated via an online calculation engine powered VMD, PyMOL and Matlab where protein chains from different ‘MODEL’ cards in the PDB file are combined and unique identifier (e.g. A, B, C, etc.) is assigned for each chain. In addition, the autoupdate component automatically collects the newly released PDB structures to generate results and update *i*GNM 2.0 using the offline calculation engine.

The architecture of *i*GNM 2.0 is sketched in Figure 4 and detailed in its caption. Further information about technology used in visualization module can be found in the Supplementary Text S1. We want to particularly mention that our database is regularly updated by subroutines that identify newly added PDB files and GNM computations are subsequently performed by an off-line GNM engine to catch the pace of rapidly growing PDB (averaging ∼30 new structures per day in 2014).

The *i*GNM 2.0 is freely accessible at http://gnmdb.csb.pitt.edu/ (Taiwan mirror site: http://dyn.life.nthu.edu.tw/gnmdb/). An extensive tutorial on the use of the *i*GNM 2.0 database can be found in http://gnmdb.csb.pitt.edu/Tutorial.php.

## DISCUSSION AND CONCLUSION

The *i*GNM 2.0 is a significantly enhanced version of the database *i*GNM published a decade ago. The original database found broad usage and utility in investigating the equilibrium dynamics of structures deposited in the PDB. The new version will further facilitate its usage, now containing data on more than 95% of the current PDB content, in addition to offering a user-friendly interface with advanced 3D and 2D visualization and analysis capabilities. A unique feature of *i*GNM 2.0 is the accessibility of data for BAs, rather than the single chains or asymmetric units, thus providing insights into the dynamic properties of biologically functional entities. Note that the BAs may be very different from the asymmetric units both structurally and dynamically. The differences between GNM-predicted and X-ray crystallographic (PDB-reported) B-factors may originate from artifacts such as crystal contacts between replica on adjacent lattice sites of the crystal, which would lead to lower B-factors than those predicted (by the GNM) for the isolated molecule (51–53). Conversely, calculations performed for the asymmetric unit would miss the effect of inter-subunit contacts and depart from the B-factors that are reported for the BA. The subset S2B (Table 1) represents the latter case: there is a considerable improvement (from 0.513 to 0.610) in the correlation with experimental data, when the B-factors are computed for the entire BA composed of multiple subunits (and not for the asymmetric unit only, which would be retrieved as the default structure in the PDB). Exploring of the dynamics of the BA itself is therefore essential to extracting biologically meaningful results. Supplementary Figure S2 illustrates this feature for a voltage-gated sodium channel. The most collective (softest) modes of motions of BAs often underlie allosteric or large-scale cooperative rearrangements of entire subunits or domains.

In previous work, different representations have been adopted for ENM nodes, e.g. Setny and Zacharias proposed the center of the ribose sugar in the backbone to be the best site for the nucleotide ENM node (54). Good agreement with experimental data on nucleotide-containing structures (DNA/RNA and their complexes) has been obtained in *i*GNM 2.0 by adopting a 3-node representation for each nucleotide, the nodes being placed at the sugar, the base and the phosphate groups. This representation has recently proven to accurately reproduce the principal modes sampled by microseconds simulations (31).

The present structural-proteome scale analysis clearly shows that the agreement of GNM predictions with experiments improves with the size of the investigated structure. This property became clear here by performing systematic computations for large structures and BAs. A close look at the correlations with experimental B-factors, in the range N < 1400, is presented in Supplementary Figure S3 panel A for three different subsets listed in Table 1. Another feature worth noting is that the GNM usually yields more accurate results for globular structures. We can see in Panel B the decrease in correlation with increasing axial ratio.

Examination of structures of even 10^4^ residues showed that the accuracy of the results did not decrease with increasing size. In particular, we noted that the current *i*GNM 2.0 computations for the 14 PDB structures deposited to date in the PDB for various forms of the ribosome (30S, 40S or 70S subunits, complexed with different proteins) led to correlation coefficients of ∼0.7 with experimental data on residue fluctuations (see Supplementary Figure S4) in addition to indicating slow modes and cross-correlations consistent with experimentally observed rachet-like mechanism.

Large structures/assemblies are actually the most challenging systems for molecular dynamics simulations, and most simulations for systems of the order of 10^3^ residues are limited to short durations, far from sampling the collective dynamics or cross-correlations that cooperatively involve the intact structures. In this respect the *i*GNM 2.0 is distinguished as a resource that provides information on the collective dynamics of this challenging set of structures.
